# Engaging the Influence of Global Private Actors in Health in Sub-Saharan Africa

**DOI:** 10.34172/ijhpm.9541

**Published:** 2026-03-10

**Authors:** Rene Loewenson, Sharifah Sekalala, Tatenda Chatikobo

**Affiliations:** ^1^Training and Research Support Centre, Harare, Zimbabwe.; ^2^School of Law, University of Warwick, Coventry, UK.

## Introduction

 Global powerful private actors (PPAs), such as private financial institutions, transnational corporations (TNCs), private (philanthropic) foundations, global accountancy and consultancy firms, and ultra-wealthy individuals contribute diverse resources for improved health. However, these actors can also negatively impact on health and constrain public health improvements.^1^ In sub-Saharan Africa (SSA), the presence of PPAs has grown, illustrated by a 66% growth of private equity and venture investments between 2017-2022. Currently, TNCs play a key role in mineral extraction, agribusiness and digital technologies, and control the bulk of SSA’s US $12.7 billion pharmaceutical market.^1-3^ Beyond their direct health impact, PPAs exert significant financial influence. For instance, the top 5 private foundations in SSA, including the Bill and Melinda Gates Foundation, have made major investments in commercial processes and new digital technologies that amplify their role in health systems.^2^

 Measuring the direct health impacts of these PPAs is important. However, exposing and understanding the pathways through which they influence health policy and decisions on the continent is as important. Such understanding can inform engagement to protect and promote public health in SSA, including for more disadvantaged communities.

 As background to this viewpoint, we explored the pathways of PPA influence on health in SSA through a desk review of over 219 public domain documents. The review covered five purposively selected areas of PPA activity—food, essential medicines, extractive industries, information and finance—that impact health in SSA.^2^ As noted in the previous paragraph, these are sectors of historical or significant growth in transnational and PPA activity. While the five areas are not exhaustive of all areas of transnational and PPA activity, they have been purposively selected for their scale, growth and relevance for health. The searches and data capture were implemented by all authors using as search terms “Sub-Saharan Africa” and each of the five areas indicated of PPA activity and “health” or “policy” or “power” or “politics” or “economy” or “accountability,” and with inclusion of papers published from 2000 onwards. The findings suggest entry points for policy dialogue and action to promote public health in the interactions with PPAs in SSA.

## Pathways of Powerful Private Actor Influence on Health

 We found diverse pathways at different levels of the influence of PPAs.

###  A Neoliberal Context Drives the Power of Powerful Private Actors 

 The dominant global neoliberal policy in SSA and post-Cold War globalisation shrunk the state and weakened market regulation. Economic reforms led to the “rolling-out” of private investment and the introduction of new public management principles. As exemplified in Table, neoliberal policy reforms opened space for and strengthened the structural, agential, and narrative forms of power of PPAs in health-impacting sectors.^1^

**Table:** Neoliberal Drivers of Powerful Private Actor Influence in Health

**Sector**	**Neoliberal Drivers of Influence**
Food systems	A Green Revolution agribusiness model, supported by private foundations, has expanded corporate land acquisition, displaced local food producers and their knowledge, and intensified corporate inputs to farming and food imports, including of ultra-processed foods. The changes in food systems have been associated with a rise in obesity, undernutrition and chronic disease.^1,2,5-7^
Mining sector	PPA mineral extraction claims to bring significant economic contribution to SSA, but in a neoliberal model of “enclave” processes, using imported equipment, technical, financial and managerial services and with limited value-addition processing in SSA. This limits economic returns in SSA, while extraction also displaces communities, degrades environments and impacts on worker and community health, adding to inequities in health. Deregulation in liberalisation policies promote less effective voluntary measures over legal controls, including to manage health impacts.^1,2,5,8^
Finance sector	Funding conditionalities disincentivise investment in public systems, including health, and set investment requirements that privilege TNCs. Global tax systems limit tax revenue in SSA in favour of TNC headquarter countries and tax havens. Circular capital flows between large private foundations and for-profit companies consolidate PPA power and raise concerns on conflict of interests.^1,2,4,9,10^
Pharmaceutical sector	TNC domination of the medicines market and control over medicine prices adds to investment terms in significant funding from global and private foundations in the sector. Prequalification requirements, a global funder preference for TNC suppliers, and WTO IP rules disincentivise local medicine production compared to TNC imports, affecting distributed and equitable access to medicines and other health technologies in SSA.^1-3^
Information/digital systems	Private equity investment in TNC digital services (“Big Tech”), private foundation funding of their role in large language models and artificial intelligence in health and WTO IP rules give PPAs advantage over African corporations in digital technologies and infrastructures, contributing to digital inequalities and their role in health.^2,3,7^

Abbreviations: WTO, World Trade Organization; IP, intellectual property; PPA, Powerful Private Actor; SSA, sub-Saharan Africa; TNC, transnational corporation.

###  Powerful Private Actors Exert Narrative and Agential Influence 

 A neoliberal policy context has sustained narratives that “private is best,” leading to distrust of local alternatives and public systems. This is illustrated, for instance, in questioning of the standards of locally produced medicines, or critiquing national health insurance efforts as negatively impacting on investor confidence. Pro-market rhetoric has blamed local capacity shortfalls for high medicine prices, rather than a prohibitive intellectual property (IP) regime.^2^ PPAs have controlled the scope of information used in policy negotiations, promoting “non-decisions” on issues brought by African countries, such as IP waivers for essential health technologies or narrowing the understanding of non-communicable disease causation.^11^ Private foundations have exerted narrative power in framing social challenges as needing market-based or TNC social responsibility solutions.^11^ As a form of agential power, PPAs have sponsored decision-makers, conferences, scholarships, and public relations campaigns. When added to their financing of health services, this has given private foundations a seat in health policy forums in SSA.^1,5,7^

###  Tax Concessions and Weakened States Strengthen Powerful Private Actor Roles 

 Weak state tax capacities in SSA, significant tax concessions given to TNCs, cross-border corporate tax abuse, strong TNC accounting and tax avoidance practices have limited tax revenue in SSA, with cascading impact on health systems and investments.^2,4,10^ In the face of weakened public revenues and rising health security and universal coverage demands, PPAs are positioned as relieving the pressure on cash-strapped SSA public services. Situating TNCs as service and technology innovators raises pressures on states in SSA to “de-risk” such private investment. PPA contributions do bring resources, but may also bring health risks and raise concerns for health equity.^1,2^ Poorly regulated digital investments by PPAs can, for example, accelerate the private extraction and control of health data, even while national statistical offices in SSA struggle to meet international reporting requirements.^1,2,12,13^

###  Representation Deficits Weaken Sub-Saharan Africa Global Influence 

 Global-level dynamics compound drivers of PPA influence *within* the continent. Global standards can help SSA governments regulate activities affecting health. However, SSA countries face representation deficits in key platforms such as the Organisation for Economic Co-operation and Development tax forums, or in inadequate delegate sizes for the United Nations (UN) forums. Such representation deficits can lead SSA governments to accept unfavourable conditions, such as in trade and IP rules, ultimately benefiting PPAs.^1,2,14^

###  Marginalising Social Voice Weakens Public Interest Alliances 

 Civil society in SSA can act as a watchdog of conflicts of interest between states and PPAs. However, this role is weakened when social participation in policy interactions with PPAs is reduced to tokenistic forms.^15^ States’ avoidance of conflict with PPAs and exclusion of civil society from negotiations have generated public distrust. Rather than building public-public interest alliances to address power imbalances with PPAs, such distrust has engendered protest and litigation over PPA processes, such as on farmer displacement in extractive activities, or on vaccine pricing in trade agreements.^1,2,6,8^

## Pathways of Influence Are Entry Points for Response

 While these multiple drivers of PPA influence in health in SSA may present power asymmetries in public health decision-making, they also present areas to engage both *within* SSA and at the global level, including by building on initiatives underway.

###  Generating Evidence and Narratives to Strengthen Public Health Interest 

 Public health action in SSA depends on exposing, engaging with and, where relevant, litigating on harmful outcomes and conflicts of interest. SSA states, civil society and technical actors have increasingly exposed harmful practices, including in campaigns on access to information, public evidence on TNC tax and financing flows, or in civil society petitions over International Monetary Fund debt-financing arrangements. These actions have challenged false narratives and communicated public health evidence, including through investigative journalism.^2^ While ad-hoc research has generated important evidence, the scale of PPA influence calls for a more institutionalised production and public domain reporting of evidence, including to expose social inequalities in impact. Examples of this are legislation on PPA disclosure, on reporting duties and on conflicts of interest, and mandating Health Impact Assessment of health-impacting commercial activities, as has been done for Environmental Impact Assessment.^2^

###  Shifting From Voluntary to Legal Measures 

 Engaging with PPA influences calls for institutional, procedural and legal changes in areas where there are harms to health. National standards enable a move from voluntary to legal measures in health, while there is effort to set harmonised continental and sub-regional standards to avoid a competitive “race to the bottom” between countries. The resolutions of the African Commission on Human and Peoples’ Rights include provisions to protect health rights in areas of private actor involvement in health.^16^ Regional initiatives have engaged on harmonising health duties of TNCs, such as on compensation for chronic disease in ex-mineworkers, and on inclusion of prior informed consent by communities on agreements with TNCs in mining.^8^ Rather than seeing regulation as a barrier, SSA pharmaceutical manufacturers see unified regional regulation as a boost to their manufacturing and have created an African Medicines Agency.^17^

 A robust civil society, investigative journalism and legal protections on freedom of information provide levers for accountability on rights and duties, and promote their enforcement. PPA rights breaches have already led to litigation, such as over harms to health and its determinants of the oil pipeline development in East Africa; or over disclosure of COVID-19 vaccine agreements between the state and TNCs in South Africa.^1,2^ Intensifying public-public interest alliances to strengthen enforcement calls for joint societal, state and regional monitoring and data sharing, public interest oversight and whistle-blower protection. Off-budget funding in public-private initiatives and regulatory measures should be returned to parliamentary and public domains for democratic scrutiny.

###  Widening Debate and Action on Economic Alternatives

 The pathways for PPA influence point to a “bottom line” of measures that engage with the neoliberal context that enable the power of PPAs. Such measures draw from economic policy alternatives that balance social, economic and ecosystem needs, and particularly those that integrate improved health in economic activity. For example, the UN Economic Commission for Africa has called for accelerated efforts and accountability in improving social and environmental returns from the mining sector.^18^ The African Union and SSA finance ministers have explored measures to stop trade- and finance-related leakages, have set harmonised tax laws to avoid a “race to the bottom” and established a unified continental African Tax Administrative Forum to improve tax capacities.^2,10^

 Within SSA, domestic producers have already generated economic alternatives and called for treaty negotiations to ensure the policy latitude to adopt or reject technologies and protect health and the environment.^1^ The Alliance for Food Sovereignty in Africa, representing more than 200 million African farmers and other communities, advocates an agroecology model based on farmers’ rights to choose seeds and methods of cultivation, supported by investment in local food production, short supply chains and climate-resilient and ecologically-sustainable farming.^2,7^

 Stimulated partly by the COVID-19 pandemic, the Africa Centres for Disease Control and Prevention and the African Development Bank have promoted a digital health transformation strategy to support locally-driven digital health innovations in SSA.^19^ Such strategies, technology hubs, and other forms of continental investment can complement state mechanisms that reach the community, such as through procurement tenders, tariffs, taxes, investment, and innovation funds that incentivise health-promoting domestic production alternatives in key sectors of PPA activity.

###  Securing African Health and Democratic Accountability at Global Level

 The global nature of PPAs and their influence calls for global engagement, building on continental collaboration and alliances, such ason IP, tax rules, and UN reform. This existing African initiative can extend to other areas where PPAs impact on health, such as food sovereignty, or bringing an African lens into key UN Human Rights instruments, including the Council Guiding Principles on Business and Human Rights.^20^

 Proactive global engagement implies strengthening unified continental negotiations and platforms, such as the African Tax Administrative Forum, the Africa Group of diplomats, the African and regional economic communities, supported by African digital resources. The value of these platforms is evident in the successful SSA government, diplomat and civil society promotion of a UN resolution adopted in December 2022 to bring global tax dialogue under the auspices of the UN. Their contribution to African agency is also evident in African efforts to overcome the IP constraints in the World Trade Organization (WTO) Trade-Related Aspects of Intellectual Property Rights (TRIPS) Agreement to enable equitable access to medicines and health technologies around HIV and in the COVID-19 pandemic, amongst other global negotiations.^1,2,3,7^

 In the face of procedural and resource barriers and pressures to accept less favourable terms in global processes, negotiations demand collective efforts and persistent attention, including on the implementation of gains attained. Shifting resource and power asymmetries to address inequities in the global architecture implies confronting disadvantageous global rule systems or democratic deficits, such as South Africa’s appeal for more inclusive African representation in international finance institutions.^1^ African participation in South-South and other alliances and multi-sector collaborative coalitions can amplify and inform African voices in global platforms in areas related to health. For this, the processes and measures in these partnerships need to provide meaningful spaces for a SSA lens and interests, especially for lower-income countries and communities.

## In Conclusion

 PPAs have multiple pathways of influence in health in SSA, through narrative, agential and structural power. Yet we have found also multiple opportunities and initiatives in SSA to identify and engage the policy, legal, information and institutional levers of the power behind this influence where this is needed to promote population health. Implementing these measures calls for strengthened and strategic leadership and governance. While focused more generally on private sector engagement, rather than PPAs specifically, Amri et al highlight, for example, the role of formalising dialogue mechanisms, aligning private actors to public goals, and strengthening strategic vision and policy design for health systems to engage and integrate equity in relations with private actors.^21^ Within the available options, it would be tempting to focus on easier measures and short-term “wins.” These options are necessary, including to build learning and confidence, but may not be sufficient. A current global context of poly-crisis, conflict and inequality requires deeper actions to dig at the roots of the rule systems, regimes, and measures that both raise health risks and health inequities and give PPAs the structural power to limit more self-determined public interest action in SSA to control these risks. This is important to build the political economy alternatives in SSA that more sustainably and equitably promote and protect health and well-being.

## Acknowledgements

 The authors acknowledge selected references and review of drafts of the background documents from Tiffany Ansari and David McCoy of the United Nations University-International Institute for Global Health (UNU-IIGH) and Attiya Waris. The authors appreciate the contribution to ideas and discussions on global private actors in health at the Expert Group Meeting on the accountability of PPAs in global health in 2023.

## Disclosure of artificial intelligence (AI) use

 Not applicable.

## Ethical issues

 Not applicable.

## Conflicts of interest

 Authors declare that they have no conflicts of interest.

## Disclaimers

 The views expressed are those of the authors and not of the UNU-IIGH or any reviewers.
